# Dietary Patterns and Hypothyroidism in U.S. Adult Population

**DOI:** 10.3390/nu16030382

**Published:** 2024-01-28

**Authors:** Dana Alkhatib, Zumin Shi, Vijay Ganji

**Affiliations:** 1 Human Nutrition Department, College of Health Sciences, QU Health, Qatar University, Doha P.O. Box 2713, Qatar; da1602675@qu.edu.qa (D.A.); zumin@qu.edu.qa (Z.S.); 2 Department of Nutrition and Dietetics, School of Health & Human Sciences, Indiana University Indianapolis, 1050 Wishard Blvd, Indianapolis, IN 46202, USA

**Keywords:** hypothyroidism, thyroid, dietary patterns, factor analysis, NHANES, USA

## Abstract

The thyroid gland produces hormones that are essential for various body functions. Hypothyroidism is defined as insufficient thyroid hormone production. Several studies have found associations between specific micronutrients and overall thyroid function; however, the amount of evidence regarding the relationship between dietary patterns and hypothyroidism among the U.S. population is limited. Data from three cycles of National Health and Nutrition Examination Surveys (NHANES), 2007–2008, 2009–2010, and 2011–2012, were used (*n* = 8317). Subjects with serum thyroid stimulating hormone >4.5 mIU/L or on levothyroxine were considered to have hypothyroidism. Age, sex, race/ethnicity, body mass index, and several lifestyle factors were considered as covariates. Three patterns were extracted using factor analysis. These were labeled as fat–processed grains–sugars–meats (FPSM), oils–nuts–potatoes–low-fat meats (ONPL), and fruits–whole grains–vegetables–dairy (FWVD) patterns. In a weighted multiple logistic regression, FPSM and ONPL were inversely associated with hypothyroidism (OR, 0.75; 95% CI, 0.57–1; *p* = 0.049 and OR, 0.81; 95% CI, 0.67–0.97; *p* = 0.025, respectively). However, FWVD demonstrated no association with hypothyroidism (*p* = 0.63). In conclusion, FPSM and ONPL patterns but not FWVD patterns were associated with hypothyroidism in U.S. adults. Nutrient deficiencies and their interactions may be linked to hypothyroidism.

## 1. Introduction

Hypothyroidism is a common chronic disease that affects the thyroid gland. It affects about 5 out of 100 adults in the U.S. [1]. It is characterized by a deficiency in thyroid hormones, i.e., thyroxine (T4) and triiodothyronine (T3) [2], which mainly affect energy metabolism. Hypothyroidism is associated with several infirmities, such as cardiovascular diseases, type-2 diabetes mellitus, neurological symptoms, infertility, and obesity [2,3]. Symptoms of hypothyroidism are fatigue, weight gain, constipation, increased cold sensitivity, increased serum cholesterol, thinning hair, bradycardia, depression, irregular menstrual cycles, and sometimes goiter [4]. Hypothyroidism can be a result of several causes, such as Hashimoto thyroiditis, thyroidectomy, congenital thyroid malfunctions, radiation, or the use of certain medications [5]. Additionally, several minerals and vitamins are associated with thyroid hormone metabolism [6], including iodine, selenium, iron, zinc, and vitamin B-12 [7]. 

Iodine deficiency is the main cause of hypothyroidism in iodine-deficient geographic areas [8]. Iodine is needed by the thyroid gland to produce thyroid hormones [9]. Iodine can be obtained from foods, salt seasonings, and supplements. Seaweed, fish and seafood, iodized salt, and dairy products are the richest sources of iodine [10]. A study that was conducted in Sri Lanka by Abeysuriya et al. [11] found that children who consumed non-iodized salt had significantly higher thyroid-stimulating hormone (TSH) concentrations compared to those who consumed adequate iodized salt (*p* < 0.001). However, it appears that not only does iodine deficiency contribute to the development of hypothyroidism, but excess iodine might also impair thyroid function [12], given that in some populations, iodine consumption is greater than recommended [13]. Excessive iodine intake might cause iodine-induced hypothyroidism through the Wolff–Chaikoff effect mechanism [12,13]. At high iodine exposure, thyroid hormone production and release is temporarily inhibited because of the downregulation of a sodium iodide symporter protein, which is responsible for actively transporting iodine from the blood into thyroid follicular cells [14]. Thus, the thyroid gland will not be able to effectively take up and utilize iodine for hormone production. Generally, this effect is transient, as the thyroid gland resumes its normal function once iodine concentrations drop back to normal [15]. However, in some cases, the Wolff–Chaikoff effect persists, causing long-term hypothyroidism [15]. 

Many studies have linked diet to the etiology of various diseases, including cardiometabolic and autoimmune diseases and psychological disorders [14,15,16]. Because individuals do not consume a single or limited number of nutrients, but consume different types of foods, it is important to study the whole diet rather than a single nutrient to obtain a holistic overview of what individuals consume. Limited studies were conducted on the association between dietary patterns and hypothyroidism, including the link between iodine intake and thyroid function. In two experimental studies on rats, the investigators found that high animal fat intake was significantly associated with impaired thyroid function, which led to hypothyroidism, as evidenced by the increased concentrations of TSH and decreased T4 [17,18] Moreover, a cross-sectional study that was conducted in Greece [19] found that inadequate fruit and vegetable consumption was associated with higher total oxidative status, which may affect thyroid function adversely.

Studies on the relationship between the Mediterranean diet (MeDiet) and thyroid function have yielded mixed findings. An Italian study [20] found that increased MeDiet adherence was associated with decreased thyroid function. In the other study [21], healthy controls and a Hashimoto’s patients group differed in their food patterns and behaviors. The healthy controls had a more plant-based dietary pattern that was close to the MeDiet, while the other group had a more animal-based diet that included processed meat, saturated fats, and refined sugars, like the Western dietary pattern. To date, there are no studies published on the association between dietary patterns and hypothyroidism in the U.S. adult population. The existing research has predominantly examined single nutrients or other thyroid diseases, primarily thyroid cancer, rather than specifically investigating the association between dietary patterns and hypothyroidism in the U.S. adult population. The current body of literature has largely overlooked the research question concerning how dietary patterns are related to hypothyroidism in the adult population of the United States. Therefore, the objective of this study was to investigate the relationship between dietary patterns and hypothyroidism using nationally representative sample surveys in the U.S.

## 2. Materials and Methods

### 2.1. Study Design and Setting

In this cross-sectional study, data from 3 cycles of the National Health and Nutrition Examination Survey (NHANES), 2007–2008, 2009–2010, and 2011–2012, were used. NHANES is conducted by the National Center for Health Statistics of the Centers for Disease Control and Prevention. It is a program based on survey research that collects data that can be used for multi-purposes, such as assessing the prevalence of common diseases, as well as many risk factors associated with them, and to assess the association between nutritional status and health. It is also used in conducting epidemiological studies, whereby the results can be used to develop policies and health programs. 

NHANES collects data on non-institutionalized participants aged >2 years old, representing the U.S. population. Since 1999, NHANESs have been conducted periodically, every two years, to assess the health status of the U.S. population. The survey gives the opportunity to follow up with the subjects and track any changes that occur. Three different assessment tools, namely, laboratory tests, physical examinations, and interviews, are included in these surveys. NHANES follows a stratified, multistage, probability clustering in its sampling design. It also oversamples some subgroups in the population, such as minorities, children, adolescents, and older persons, for precision and accuracy. NHANES gives information about the demographics of the population, as well as their socioeconomic, health, and dietary status. These data were collected using different methods, including nurse-administered questionnaires, direct interviews, and physical examination. Further details of the sampling design and methods are described in the NHANES Survey Methods and Analytic Guidelines [22]. 

Institutional review board approval was obtained for all the conducted procedures, and all the participants submitted written informed consent that clearly explained the study design and procedures (protocol #2005-06 and protocol #2011-17).

### 2.2. Study Population

**Figure 1:**
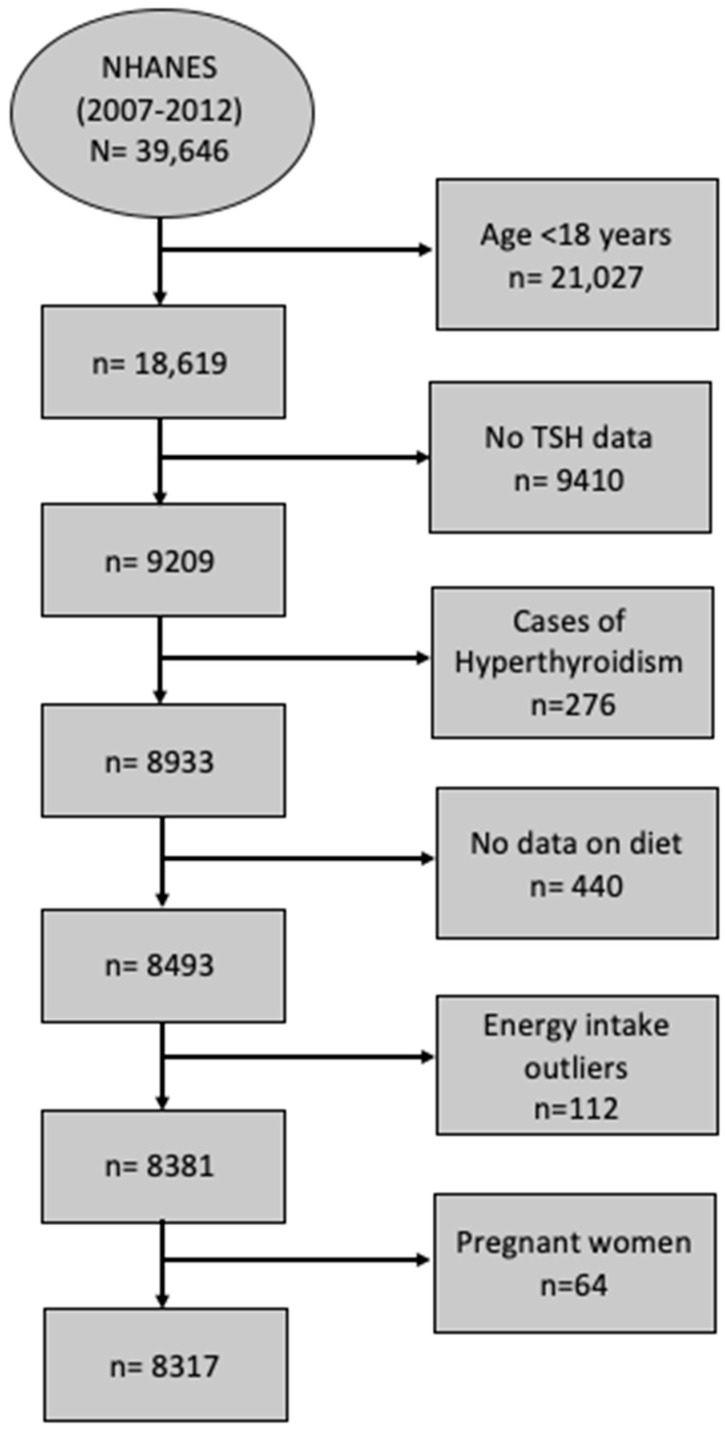
STROBE flow chart for sample derivation. Data from three cycles of NHANES, 2007–2008, 2009–2010, and 2011–2012, were concatenated into one analytic data file, NHNAES 2007–2012. The sample consisted of subjects with TSH concentration >0.4 mIU/L. Men whose intakes ≤500 kcal/day and ≥6000 kcal/day and women whose intakes ≤500 Kcal/day and ≥5000 kcal/day were considered outliers. Abbreviations: NHANES, National Health and Nutrition Examination Survey; TSH, thyroid-stimulating hormone.

In this study, data from 3 cycles of NHANES 2007–2008 (12,943 participants), NHANES 2009–2010 (13,272 participants), and NHANES 2011–2012 (13,431 participants) are consolidated into one analytic data file (2007–2012), while accounting for the appropriate survey design and weight. In NHANES (2007–2008), 10,149 people were interviewed at home, and 9762 were examined in the mobile examination center (MEC) [23]. The MEC is a portable medical unit used to conduct various tests, examinations, and measurements to conveniently gather data from participants from different locations across the country. In NHANES (2009–2010), 10,537 people were interviewed at home, and 10,253 were examined in the MEC [24]. In NHANES (2011–2012), 9756 people were interviewed at home, and 9338 were examined in the MEC [25]. All the cycles had high response rates of more than 75%. After combining the 3 cycles, the NHANES (2007–2012) dataset had 39,646 screened participants. Participants were excluded if they were ˂18 years old (*n* = 11,823), had missing TSH data (*n* = 9410), had confirmed hyperthyroidism (*n* = 276), had missing dietary intake data (*n* = 440), were shown to be energy intake outliers (men with intakes ≤500 kcal/day and ≥6000 kcal/day and women with intakes≤ 500 kcal/day and ≥5000 kcal/day) (*n* = 112), or were pregnant (*n* = 64). The final analytic sample size was 8317 (Figure 1).

### 2.3. Dietary Intake Assessment

What We Eat in America (WWEIA) is the dietary interview component in NHANES (2007–2012). WWEIA is a partnership between the U.S. Department of Agriculture (USDA) and the U.S. Department of Health and Human Services. To assess the dietary intake of the participants, detailed data regarding the previous 24 h dietary intake were collected by well-trained interviewers. All the participants were eligible for two 24 h recall interviews. The first interview was conducted face to face in an MEC, while the second one was conducted via telephone 3 to 10 days after the initial one. For quality purposes, only the first 24 h recall was used in this study. The data from the 24 h recalls were collected using the USDA’s dietary data collection instrument called the automated multiple pass method (AMPM). The AMPM consists of 5 steps: quick list, forgotten foods, time and occasion, detailed cycle, and final probe [26]. The AMPM is a validated tool and is updated every two years. For quality control and assurance, all the interviewers received condensed training and retraining sessions. Around 5% of the total interviews of each interviewer were monitored, whether via direct observations or audio recordings. The interviews were assessed twice for missing data and unclear notes. 

The dietary data collected for each participant from the 24 h interview were sent to the USDA to be converted to portion sizes, which were then converted to grams. Based on the nutrient profile of the food item, it is classified into a certain food group. After the data was processed, it was made available to the public for research purposes. The dietary data were aggregated into 28 food groups according to the modified Food Patterns Equivalents Database [27] (Table 1). The 28 food groups were used for dietary pattern construction. The dietary patterns were labeled based on the factor loading scores, which represent the correlation coefficients between the dietary patterns and identified food groups.

### 2.4. Biochemical Measurements

In the current study, participants with TSH > 4.5 mIU/L or on medication for hypothyroidism (levothyroxine) were considered to have hypothyroidism regardless of whether it was overt or subclinical. The serum TSH concentration was quantitatively determined using a two-site immune-enzymatic access assay. Further details can be found in the laboratory procedure manual [28].

### 2.5. Other Measurements

The association between the variables of interest can be confounded by several covariates. Models were constructed to adjust for the common variables that are known to possibly affect this association. The covariates were either socioeconomic characteristics or known risk factors. Age, sex, race-ethnicity, body mass index (BMI), physical activity, smoking status, alcohol consumption, education, poverty–income ratio, and daily energy intake were considered as covariates. Participant demographics, including age, race, education, income, and physical activity were self-reported by the participants during house visits and interviews, which were performed by trained interviewers with the use of the computer-assisted personal interviews system. Height and body weight were assessed using standardized methods [29]. Height was measured using a stadiometer, and weight using a standard digital scale; these were measured in ideal standard conditions. BMI was calculated from the obtained height and weight, as kilograms of body weight divided by meters of height squared (kg/m^2^). According to the metabolic equivalent task (MET), physical activity was classified as mild (˂600 METs), moderate (600–1200) METs, and intense (≥1200 METs). The physical activity questionnaire (PAQ) was administered to determine the level of physical activity among the participants. The PAQ is a self-administered questionnaire that gathers data on habitual activities, as well as physical activity during work and transportation. The questionnaire includes questions regarding the duration, intensity, and frequency of various activities. It is worth noting that the PAQ is based on a standardized questionnaire developed by the World Health Organization, named the Gold PAQ [30]. Smoking status was defined as being a current smoker, former smoker, and non-smoker. Alcohol consumption was categorized into drinkers, non-drinkers, and unreported. Education level was categorized into <11 grade, high school or general education development, college graduate, and post-graduates. The poverty-to-income ratio (family income/family poverty threshold) was categorized as low (<1.3), moderate (1.3–3.5), and high (>3.5). The participants could choose to do the interview in either English or Spanish or to have an interpreter.

### 2.6. Statistical Analysis

STATA’s statistical software package was used to analyze the data (version 17, Stata Corp LLC, College Station, TX, USA). Complex survey design and clustering were accounted for during the analyses. A descriptive analysis was performed for all the data for subject characteristics. The demographics and socioeconomic variables were compared between the subjects with hypothyroidism and others with euthyroidism. Continuous variables are presented as means and standard deviations, whereas categorical variables are presented as frequencies and proportions (%). To compare the subjects’ characteristics, the ANOVA was used to compare the differences between the continuous variables, and the chi-square test was used to compare the differences between the categorical variables. 

An exploratory factor analysis was performed to construct dietary patterns, using the 28 food groups. The number of patterns extracted was based on three determinants: scree plot, eigenvalue, and the interpretability of the factors. The consumption of different foods by each individual was used to calculate a score for each factor, which was subsequently used to estimate the level of consumption of that particular factor by the individual. Eigenvalues measure the total variance in the variables explained by the common principal component. The factor with the highest eigenvalue score is considered the principal component. Accordingly, considering the interpretability of the factors and the scree plot, factors with an eigenvalue >1.3 were considered. To determine the uncorrelated factors and to reduce the number of indicators that have high loading on one factor, we used the principal component analysis method for factor extraction and varimax rotation. We used the FACTOR function present in the STATA program. Varimax rotation is considered an orthogonal rotation that is used to maximize factor variance. Each food group was given a factor loading score to aid in categorizing food groups into three food patterns. A factor loading score of more than 0.3 was used to determine the food groups that mostly contribute to the food pattern. Factors were labeled as dietary patterns based on the factor loading scores of the foods.

A weighted multiple logistic regression was performed to calculate the odds ratio (OR), with a 95% confidence interval (CI), to determine the associations between dietary patterns as continuous variables and the risk of hypothyroidism. The dietary patterns were treated as continuous variables to maximize the statistical power of the analyses. Three regression models were adjusted for various covariates. Model 1 was adjusted for age, sex, and energy intake. Model 2 was adjusted for age, sex, energy intake, race, education, income, smoking, alcohol consumption, and physical activity. Model 3 was adjusted for age, sex, energy intake, race, education, income, smoking, alcohol consumption, physical activity, and BMI. The results are considered statistically significant if *p* < 0.05. 

## 3. Results

### 3.1. Study Sample Characteristics by Hypothyroidism Status

The characteristics of the study sample by hypothyroidism status are presented in Table 2. The sample consisted of 50.7% men (*n* = 4220) and 49.3% women (*n* = 4097). Ninety-one percent of the total subjects had normal thyroid function (*n* = 7555) and nine percent had abnormal thyroid function (*n* = 762). Most of the subjects with abnormal thyroid function were women (67.5%). Of the 8317 subjects, 46.3% were non-Hispanic white (*n* = 3851), 19.9% were non-Hispanic black (*n* = 1655), 16.4% were Mexican American (*n* = 1365), and 17.4% were of other ethnicities/races (*n* = 1446). Of the total sample, 21.2% (*n* = 1677) self-reported as being current smokers, while 28.6% of the participants were not high school graduates (*n* = 2262). The education level was not significantly different between the normal and hypothyroidism groups (*p* = 0.43). Around 22% of the subjects with normal thyroid function were smokers, while 14.9% of those with abnormal thyroid function smoked. Alcohol consumption was more common in the normal group (60.9%) compared to the hypothyroidism group (55.2%). BMI was significantly higher in the hypothyroidism group (29.9 vs. 28.7 kg/m^2^; *p* ˂ 0.001). The normal thyroid group had higher physical activity compared to the hypothyroidism group in terms of MET/wk (*p* ˂ 0.001). Hypertension was significantly higher in the hypothyroidism group compared to the normal group (*p* ˂ 0.001).

### 3.2. Dietary Intake Patterns Description

The factor loading scores and variances explained by each food pattern are presented in Table 3. Three major food patterns were determined based on the factor analysis method using 28 predefined food groups. The factor loading scores determined how much each food group contributes to each factor. Positive factor loading scores mean that the variable/food group positively correlates with the principal component. The higher the positive loading, the greater the correlation, which means that this variable has a strong impact on that factor. However, negative loadings indicate a negative correlation between the variable and the factor, and that it contributed the least to this component. 

**Figure 2:**
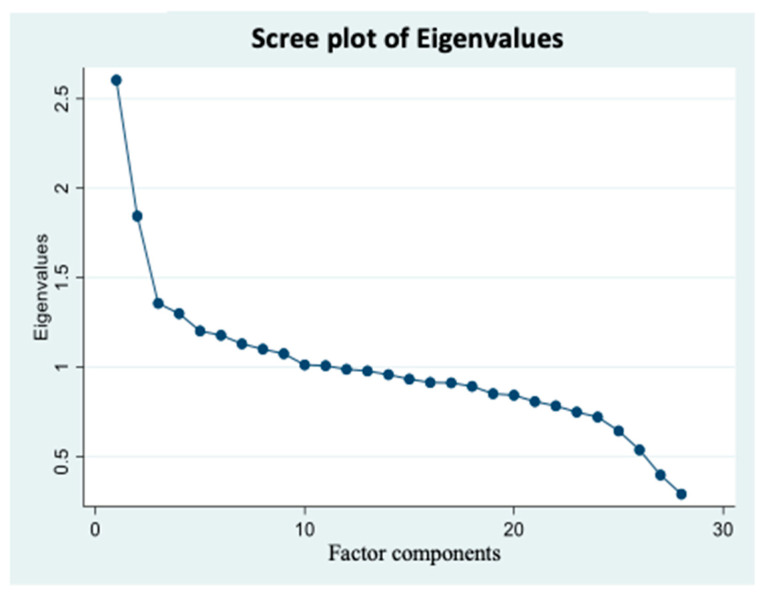
Scree plot of Eigenvalues based on the pre-determined 28 food groups’ intake frequency. Factor components are on the X axis, and their corresponding eigenvalues are on the Y axis and potential factors are on the X axis. Graph represents continuum of eigenvalues. Each dot represents a potential factor. A scree plot was used to visualize the number of factors that can be extracted. Factors with ≥1.3 eigenvalues were considered. Accordingly, three factors were extracted in the factor analysis. Data came from three cycles of National Health and Nutrition Examination Surveys 2007–2012 (*n* = 8317).

According to the scree plot (Figure 2), all the scores with an eigenvalue ≥1.3 were considered as possible factors. Three factors were extracted. The labeling of food patterns was predominantly based on the factor loadings of foods within each pattern. The factor loadings indicate the contribution of each food in shaping the overall pattern, ensuring that the pattern labels effectively reflect the key components and features of each food pattern. It is worth acknowledging that pattern labeling is of a subjective nature. The first factor had high factor loading scores for solid fats, refined whole grains, cheese, added sugar products, processed meats, tomatoes and tomato products, and oils. Thus, this factor was labeled as the fat–processed grains–sugars–meats (FPSM) pattern. The second factor had high factor loadings for oils, nuts and seeds, potatoes, vegetables, chicken, turkey, eggs, seafood, and dark-green vegetables. Therefore, it was named as oils–nuts–potatoes– low-fat meats (ONPL) pattern. The third factor had positive factor loading scores for fruits, whole grains, vegetables, yogurt, milk, soy products, and legumes. Thus, it was labeled as the fruits–whole grains–vegetables–dairy (FWVD) pattern. Factor 1 was the most dominant dietary pattern. Indeed, most variance was explained by the FPSM pattern (9.3%), while the least variance was explained by the FWVD pattern (4.8%). The ONPL pattern explained 6.6% of the variance. A total of 20.7% variance was explained by all three dietary patterns, which is similar to the published reports [31].

### 3.3. Association between Food Patterns and Hypothyroidism

The associations between the three dietary pattern scores (used as a continuous variable in the model) and the risk of hypothyroidism are presented in Table 4. No significant association was observed between the dietary patterns and hypothyroidism after adjusting for age, sex, and energy intake (model 1). The ONPL pattern was significantly associated with a lower risk of hypothyroidism after further adjusting for the additional covariates in model 2 (OR 0.83, 95% CI: 0.69–0.99, *p*-value = 0.037). After further adjusting for BMI in model 3 (a multivariate-adjusted model), the significant association between the ONPL pattern and hypothyroidism persisted (OR 0.81, 95% CI: 0.67–0.97, *p*-value = 0.025). Moreover, the FPSM pattern was significantly associated with a lower risk of hypothyroidism in the multivariate-adjusted model (OR 0.75, 95% CI: 0.57–1, *p*-value = 0.049), but the association was not as strong as the one between the ONPL pattern and hypothyroidism. The FWVD pattern showed no significant association with hypothyroidism, even after controlling for all the covariates in the fully adjusted model (OR 1.03, 95% CI: 0.91–1.16, *p*-value = 0.63).

## 4. Discussion

To our knowledge, this is the first study to investigate the association between different dietary patterns and hypothyroidism risk among the U.S. population using NHANES data. Three dietary patterns were extracted, namely, FPSM, ONPL, and FWVD. We found that the FPSM and ONPL patterns were significantly associated with a lower risk of hypothyroidism. ONPL has a stronger association as compared to the FPSM pattern. The FWVD pattern was not associated with hypothyroidism risk.

FPSM had high factor loadings for solid fats, refined whole grains, cheese, added sugar products, processed meats, tomatoes and tomato products, and oils. As per the current literature, the FPSM dietary pattern is similar to the Western diet in its characteristics. The negative effects of the Western diet on health status are well documented, and it is associated with several health conditions [32,33,34]. In this study, the FPSM pattern surprisingly showed protection against hypothyroidism. This finding can be due to several reasons. FPSM is high in meat products that have high contents of iron, zinc, and selenium, which are all found to be associated with improved thyroid function [35]. Moreover, animal products tend to be richer in iodine compared to plant-based products [36]. Iodine is the key nutrient for thyroid hormone synthesis, so its deficiency is directly linked to impaired thyroid hormone synthesis [37]. Additionally, meats are one of the main sources of vitamin B-12 [38], and its deficiency has been found to be associated with hypothyroidism. A study by Kalicanin et al. [39] found that healthy controls had higher consumption of red meat compared to the Hashimoto thyroiditis group (OR 0.80, *p* < 0.0001). FPSM has also high factor loading for cheeses, which contain iodine and vitamin D. Cheeses contain around 39 µg/100 g of iodine, which is 26% of the iodine recommended dietary allowance (150 µg/day). However, they provide about 24 IU of vitamin D, which falls short of the recommended intake of 600 IU/day for adults, making cheeses not a rich source of vitamin D, as commonly thought. Low concentrations of vitamin D are associated with autoimmune hypothyroidism disease [40]. Even though several studies showed that high fat intake was associated with adverse health effects, including impaired thyroid function, others propose that the type of fat matters. The FPSM pattern included oils, which are high in polyunsaturated fatty acids (PUFA) and phenols that have anti-inflammatory effects and protect against oxidative stress [41,42]. Plant oils are also rich in fat-soluble vitamins, such as vitamin E, which is a potent antioxidant [43]. According to a systematic review conducted by Pang et al. [44], olive oil contains oleic acid and phenols, which are both found to support thyroid function. The effect of oils on thyroid function was also reported in a study by Kalicanin et al. [39], where they found that plant oil consumption was associated with better T3 concentrations among the Hashimoto thyroiditis group (β = 0.07, *p* < 0.0001).

Additionally, FPSM is high in solid fats, cheese, and meats, which are considered high in saturated fats. According to a recent report on a cardioprotective diet, one should limit animal and tropical oils, including palm oil [45]. Most studies recommend limited saturated fat intake due to its relationship to various diseases. However, a recent report did not support the recommendation of limiting such foods [46]. It is proposed that the effect of saturated fat, as an isolated food, does not truly reflect its effect, as it depends on the interaction between the food components that contain saturated fats with other unhealthy processed foods, such as refined carbohydrates. The study reported that a diet rich in naturally occurring saturated fats accompanied by low refined carbohydrates can have some health benefits while focusing on the quality, quantity, and degree of processing of the carbohydrates. In other words, the issue might be related to the level of processing of carbohydrates, not in fat itself [46,47]. Finally, in some studies, individuals following a Western diet tended to consume high amounts of iodized salt and had adequate iodine intake [48]. Iodine primarily comes from salt [49]. This fact can also be applied to the participants following the FPSM pattern due to similarities between the two patterns, which might also add to the reason behind FPSM protection against hypothyroidism. Despite all the possible justifications and reasons behind the protective effect of the FPSM dietary pattern, we cannot advocate following this type of diet, as the effects of the Western diet and similar diets have been extensively studied, yielding rigidly established findings on the negative impacts of Western diets and similar diets on overall health. 

The ONPL dietary pattern tended to share characteristics with the MeDiet, which is high in oils, nuts and seeds, potatoes, vegetables, chicken, turkey, eggs, seafood, and dark green vegetables. The MeDiet has anti-inflammatory and antioxidant functions, protecting against several chronic diseases. Studies on the relationship between the MeDiet and thyroid function are limited and provide inconsistent findings. The prevailing evidence on the MeDiet suggests that low MeDiet adherence is associated with an increased risk of thyroid cancer, mainly driven by inflammation and oxidative stress [19,50]. As for the individual components, several studies state that olive oil has anti-inflammatory and immunomodulatory impacts [51]. As previously discussed, many plant oils can possess anti-inflammatory effects due to the high content of PUFA, phenols, and fat-soluble vitamins, which all show positive effects on thyroid function [44]. Additionally, ONPL is high in thyroid-boosting nutrients, such as iodine, selenium, iron, selenium, vitamin B-12, and vitamin D. Specifically, nuts and seeds contain high amounts of selenium and zinc [52,53], which are needed for the metabolism of thyroid hormones. Nuts are nutrient-dense foods that are rich in several other bioactive compounds [53]. Specifically, Brazil nuts are one of the richest sources of selenium [54]. Seeds such as pumpkin, sesame, and almonds are rich in tyrosine and iodine [54], and consumption of these foods may boost thyroid function because thyroid hormones are made up of iodine and tyrosine resides.

Seafood also contributes to the ONPL pattern. Fish and seafood are rich sources of iodine, PUFA, vitamin D, vitamin B-12, selenium, iron, and zinc, whose deficiencies are associated with autoimmune thyroid disorders [55]. The iodine content of seafood varies among seafood types, ranging from 2 to 289 µg/100 g [56]. Poultry and eggs are also frequently consumed by those people who follow the ONPL dietary pattern. Poultry is not only a rich source of protein but is also rich in iodine, monounsaturated fatty acids, selenium, and zinc [57,58]. Eggs are rich in thyroid-supporting nutrients, such as iodine, vitamin B-12, iron, vitamin D, zinc, and selenium [59].

In addition, the ONPL pattern includes a variety of vegetables. Inadequate intake of fruits and vegetables is associated with higher oxidative stress [60,61]. Vegetables are high in polyphenols, which are associated with a reduced risk of autoimmune diseases [62]. People who adhere to the MeDiet have low inflammation [63]. This dietary pattern tends to be high in dark-green vegetables, which are rich in vitamins, minerals, dietary fibers, and phytochemicals, and have antioxidant properties [64]. They are also good sources of vitamin A and iron [65]. Vitamin A was also found to play a role in thyroid metabolism and homeostasis [66], whereby it increases the activity of the Na/I symporter within thyroid cells. Vitamin A deficiency is associated with altered TSH secretion, impaired iodothyronine coupling, reduced thyroglobulin synthesis, and impaired iodine uptake [66]. Additionally, the ONPL pattern is high in potatoes, which provide several crucial nutrients, such as vitamin B6, potassium, and magnesium, which support thyroid function [67]. 

On the other hand, the FWVD pattern closely resembles a lacto-vegetarian diet because it has high factor loadings for fruits, whole grains, vegetables, yogurt, milk, calcium-fortified products, soy products, and legumes. This pattern is also similar to a prudent dietary pattern. Despite the beneficial effects of vegetarian dietary patterns on health, our analysis showed no association between the FWVD pattern and hypothyroidism. In contrast to our findings, Tonstad et al. reported a significant association between a lacto-ovo-vegetarian diet and the risk of hypothyroidism [68]. It is worth noting that FWVD diet contains high amounts of goitrogenic foods (cruciferous vegetables) and milk, which have been shown to have an adverse effect on thyroid hormones [69,70,71]. The reasons for a lack of association between FWVD and hypothyroidism is not known.

The current study has several strengths. Firstly, our study was based on a nationally representative sample. Hence, the findings can be extrapolated to the population described in this study. Secondly, we have a relatively large sample size, increasing the precision of the estimate. Thirdly, the NHANES is rich in various variables, which makes it possible to construct multiple models after adjusting for several confounding variables. However, our findings have some limitations. The cross-sectional design of this study restricts causality inference. Secondly, the dietary intakes were based on a single 24 h recall, which may not reflect the habitual intakes of the participants. Recall bias is also possible. Thirdly, even though we have adjusted for the common covariates, residual confounding is still possible. The use of certain medications, mainly the ones that affect iodine metabolism (e.g., amiodarone and heparin), and the presence of patients diagnosed with other pathologies related to the thyroid, like tumors, might confound the results. Finally, thyroid peroxidase (TPO) antibodies (TPO) were not measured, affecting the accuracy of the diagnosis. TPO tests are important in differentiating between thyroid disorders, including distinguishing hypothyroidism from Hashimoto’s thyroiditis.

## 5. Conclusions

In conclusion, hypothyroidism was inversely associated with the FPSM and ONPL patterns, but not with FWVD. Restrictive diets tend to increase the risk of nutrient deficiencies. However, diets high in oils, nuts, seeds, seafood, vegetables, and low-fat protein tend to protect against hypothyroidism due to their antioxidant and anti-inflammatory properties, as well as having a low risk of deficiencies. We do not recommend FPSM as a part of a well-balanced diet. A well-balanced diet that satisfies all dietary needs may reduce hypothyroidism risk. An adequate intake of thyroid-boosting nutrients supports thyroid function. For a deeper understanding of the causal association between dietary patterns and hypothyroidism, high-quality interventional study designs are required. Personalized nutritional approaches considering genetic predisposition, metabolic differences, and gut microbiota can aid in developing tailored dietary interventions that best fit individual conditions, supporting thyroid health. Pursuing these future research recommendations can further optimize dietary interventions for better hypothyroidism management and prevention, enabling healthcare providers to offer more effective dietary interventions to individuals at risk of or already diagnosed with hypothyroidism.

## Figures and Tables

**Table 1:** Food Patterns Equivalents Database (FPED) 2011–2012 Modified Food Groups: National Health and Nutrition Examination Survey 2007–2012 (*n* = 8317). ^1, 2^.

FPED 2011–2012 Food Group	Modified FPED 2011–2012 Subgroups
Fruits	Citrus, melons, and berries Other fruits Fruit juice
Vegetables	Dark-green vegetables Tomatoes Other red and orange vegetables Potatoes Other starchy vegetables Other vegetables Beans and peas are computed as vegetables
Grains	Whole grains Refined grains
Protein foods	Meat (beef, veal, pork, lamb, game) Cured meat (frankfurters, sausage, corned beef, cured ham and luncheon meat made from beef, pork, poultry) Organ meat (from beef, veal, pork, lamb, game, poultry) Poultry (chicken, turkey, other fowl) Seafood high in n-3 fatty acids Seafood low in n-3 fatty acids Eggs Soybean products (excludes Ca-fortified soy milk and mature soybeans) Nuts and seeds
Dairy	Milk (includes Ca-fortified soy milk) Yogurt Cheese
Oils	Oils
Solid fats	Solid fats
Added sugars	Added sugars
Alcoholic drinks	Alcoholic beverages

^1^ Data from three cycles of NHANES, 2007–2008, 2009–2010, and 2011–2012, were concatenated into one analytic data file, NHNAES 2007–2012. ^2^ Food intake data were condensed into 28 food groups.

**Table 2:** Sample characteristics by thyroid status: National Health and Nutrition Examination Survey (NHANES) 2007–2012 (*n* = 8317) ^1, 2^.

Characteristics	Total ^3^	Normal ^3^	Hypothyroidism ^3^	*p*-Value ^4^
	*n* = 8317	(≤4.5 mIU/L) n = 7555	(>4.5 mIU/L) n = 762	
Energy intake, kcal	2092 ± 930	2118 ± 939	1829 ± 792	<0.001
Age, y	48.6 ± 18.6	47.5 ± 18.4	59.5 ± 17.2	<0.001
Sex				<0.001
Men, *n* (%)	4220 (50.7)	3972 (52.6)	248 (32.5)	
Women, *n* (%)	4097 (49.3)	3583 (47.4)	514 (67.5)	
Race/ethnicity				<0.001
Non-Hispanic white, *n* (%)	3851 (46.3)	3337 (44.2)	514 (67.5)	
Non-Hispanic black, *n* (%)	1655 (19.9)	1599 (21.2)	56 (7.3)	
Mexican American/Hispanic, *n* (%)	1365 (16.4)	1264 (16.7)	101 (13.3)	
Other races/ethnicities, *n* (%)	1446 (17.4)	1355 (17.9)	91 (11.9)	
Education				0.43
<11 grade, *n* (%)	2262 (28.6)	2058 (28.7)	204 (27.2)	
High school/GED, *n* (%)	1848 (23.3)	1671 (23.3)	177 (23.6)	
Some college, *n* (%)	2171 (27.4)	1973 (27.5)	198 (26.4)	
>College, *n* (%)	1635 (20.7)	1464 (20.4)	171 (22.8)	
Smoking status				<0.001
Never, *n* (%)	4231 (50.9)	3820 (50.6)	411 (53.9)	
Former, *n* (%)	2013 (24.2)	1785 (23.6)	228 (29.9)	
Current smoker, *n* (%)	1677 (20.2)	1565 (20.7)	112 (14.7)	
Not reported, *n* (%)	396 (4.8)	385 (5.1)	11 (1.4)	
Alcohol drinking (past 12 months)				<0.001
No, *n* (%)	1447 (17.4)	1278 (16.9)	169 (22.2)	
Yes, *n* (%)	5019 (60.3)	4598 (60.9)	421 (55.2)	
Not reported, *n* (%)	1851 (22.3)	1679 (22.2)	172 (22.6)	
Body mass index, kg/m^2^	28.8 ± 6.7	28.7 ± 6.7	29.9 ± 7.3	<0.001
Physical activity, METs min/wk				<0.001
<600, *n* (%)	3343 (40.2)	2949 (39)	394 (51.7)	
600–1200, *n* (%)	849 (10.2)	759 (10)	90 (11.8)	
≥1200, *n* (%)	4124 (49.6)	3846 (50.9)	278 (36.5)	
Poverty–income ratio				0.007
<1.30, *n* (%)	2424 (29.1)	2238 (29.6)	186 (24.4)	
1.3–3.5, *n* (%)	2869 (34.5)	2583 (34.2)	286 (37.5)	
>3.5, *n* (%)	2307 (27.7)	2079 (27.5)	228 (29.9)	
Not reported, *n* (%)	717 (8.6)	655 (8.7)	62 (8.1)	
Hypertension	2873 (35.6)	2496 (34.1)	377(50.4)	<0.001

^1^ Data from three cycles of NHANES, 2007–2008, 2009–2010, and 2011–2012, were concatenated into one analytic data file, NHNAES 2007–2012. ^2^ Abbreviations: GED, general education diploma; MET, metabolic equivalent task; TSH, thyroid-stimulating hormone. ^3^ Participants with TSH > 4.5 mIU/L or on medication for hypothyroidism (levothyroxine) were considered to have abnormal thyroid function. Participants’ characteristics are presented as mean ± standard deviation for continuous variables or *n* (%) for categorical variables. ^4^ Significance between normal and abnormal. The *t*-test was used for continuous variables, and the chi-squared test was used for categorical variables.

**Table 3:** Factor loadings for food intake patterns in the U.S.: National Health and Nutrition Examination Surveys 2007–2012 (*n* = 8317). ^1, 2^.

Food Group ^3^	Factor 1	Factor 2	Factor 3
Solid fats (g)	0.83		
Refined or non-whole grains	0.71		
Cheese (cup)	0.67		
Foods defined as added sugars	0.46		
Cured/luncheon meat	0.35		
Tomatoes and tomato products	0.34		0.28
Beef, veal, pork, lamb, game	0.3		
Oils (g)	0.21	0.81	
Peanuts, tree nuts, and seeds		0.54	
White potatoes (cup)		0.41	−0.35
Other vegetables		0.38	0.37
Chicken, turkey, Cornish hens		0.34	
Eggs (chicken, duck, goose)		0.27	
Seafood (finfish, shellfish)		0.26	
Intact fruits (whole or cut)			0.51
Whole grains (oz)			0.48
Other red and orange vegetables			0.38
Dark-green vegetables (cup)		0.22	0.32
Yogurt (cup)			0.29
Intact fruits (whole or cut)			0.29
Fluid milk and calcium-fortified			0.27
Soy products			0.24
Legumes computed as vegetables			0.23
Variance explained (%)	9.3	6.6	4.8

^1^ Data from three cycles of NHANES, 2007–2008, 2009–2010, and 2011–2012, were concatenated into one analytic data file, NHNAES 2007–2012. ^2^ Factor analysis method was used to extract three principal factors. Based on factor loadings of foods, Factor 1 was labeled as fat–processed grains–added sugar–meats (FPSM) pattern. Factor 2 was labeled as an oils–nuts–potatoes–low-fat meats (ONPL) pattern. Factor 3 was labeled as the fruits–whole grains–vegetables–dairy (FWVD) pattern. ^3^ Foods were grouped based on dietary intake using 24 h recall.

**Table 4:** Association between dietary patterns and hypothyroidism: National Health and Nutrition Examination Survey 2007–2012 (NHANES) (*n* = 8317).^1, 2^.

	Model 1		Model 2		Model 3	
Food Patterns ^4^	OR (95% CI)	*p*-Value ^3^	OR (95% CI)	*p*-Value ^3^	OR (95% CI)	*p*-Value ^3^
FPSM	0.91 (0.68–1.22)	0.52	0.79 (0.6–1.03)	0.076	0.75 (0.5–7.1)	0.049
ONPL	0.92 (0.77–1.09)	0.32	0.83 (0.69–0.99)	0.037	0.81 (0.67–0.97)	0.025
FWVD	1.06 (0.95–1.19)	0.3	1.01 (0.9–1.14)	0.84	1.03 (0.91–1.16)	0.63

^1^ Data from three cycles of NHANES, 2007–2008, 2009–2010, and 2011–2012, were concatenated into one analytic data file, NHANES 2007–2012. Values are presented as OR (95% CI) obtained from multivariable logistic regression. ^2^ Model 1 was adjusted for age, sex, and energy intake. Model 2 was further adjusted for race, education, income, smoking, alcohol consumption, and physical activity. Model 3 was further adjusted for BMI. ^3^ *p* < 0.05 is considered statistically significant. ^4^ Abbreviations: BMI, body mass index; CI, confidence intervals; FPSM, fats–processed grains–sugars–meats; ONPL, oils–nuts–potatoes–low-fat meat; OR, odds ratio; and FWVD, fruits–whole grains–vegetables–dairy products.

## Data Availability

Data are publicly available on the Center for Disease Control and Prevention, National Center for Health Statistics, National Health and Nutrition Examination Survey website. Data for this study came from the publicly available databases (https://www.cdc.gov/nchs/nhanes/index.htm).
